# The complete chloroplast genome sequences of *Trapa acornis* Nakano (Lythraceae)

**DOI:** 10.1080/23802359.2021.2005478

**Published:** 2021-12-09

**Authors:** Ye Yuan, Zijian Huang, Meng Shen, Ruisen Wang, Xinhua Quan, Xiangtan Yao

**Affiliations:** a Jiaxing Academy of Agricultual Sciences, Jiaxing, China; b Zhejiang University, Hangzhou, China

**Keywords:** *Trapa acornis*, chloroplast genome, Lythraceae, phylogeny

## Abstract

*Trapa* L. *(Lythraceae),* also called water chestnut, is a genus widely distributed in the Old World. With the high edible and medical values, the water chestnut has been cultivated popularly in China since the Tang and Song Dynasties. Among all cultivars, *T. acornis* Nakano is one of the most current commercial one, which grown in Jiaxing, Zhejiang province, China. However, due to the limited availability of molecular marker resources of *T. acornis*, we still have difficulty in its identification and utilization. Here, we reported the complete chloroplast genome sequence of *T. acornis*. The result demonstrated that the chloroplast genome was 155,538 bp in length, consisting of a small single copy (SSC) region of 18,275 bp, a large single copy (LSC) region of 88,492 bp, and two inverted repeat (IR) regions of 24,386 bp. The chloroplast genome contains a total of 130 genes, including 85 protein-coding genes, 37 tRNA genes, and eight rRNA genes. The phylogenomic analysis demonstrated the sister relationship between *T. acornis* and *T. bicornis*.

*Trapa* L. (family: Lythraceae), also called water chestnut, is a floating annual aquatic plant genus that is native to the subtropical regions of Asia, Europe and Africa (Chen et al. 2007). Because of the high edible and medical value, the consumption and domestication of this genus in Eurasia could be dated back to as early as the Neolithic time. (Hummel and Kiviat 2004; Adkar et al. 2014; Guo et al. 2017). *Trapa acornis*, endemic to Jiaxing, Zhejiang province, China, is one of the most current commercial species, because of its delicious taste (Bingyang et al. 1996) and unique round horns (easy to harvest) (Lim 2013). Although it is easy to distinguish *T. acornis* from other *Trapa* species in the fruit morphological traits, the phylogenetic relationships of *T. acornis* with other *trapa* species still remain unclear due to the controversial taxonomy (Takano and Kadono 2005; Chorak et al. 2019). Here we reported the complete chloroplast genome sequence of *T. acornis* to provide genetic information for the construction of phylogenetic relationships among Lythraceae.

Samples of *Trapa acornis* Nakano were collected in Jiaxing Academy of Agricultural Sciences (120.70°E; 30.86°N), and voucher specimens were deposited in Jiaxing Academy of Agricultural Sciences (voucher No. JX20190610). Genomic DNA was extracted from silica-dried leaf tissue using DNA Plantzol Reagent (Invitrogen Co. Ltd.). DNA library construction and paired-end sequencing were performed on Illumina HiSeq 2500 platform. The chloroplast genome was assembled using GETORGANELLE pipeline (Jin et al. 2018) and annotated using Geneious R9 (http://www.geneious.com) by comparing it to *T. maximowiczii* (NC_037023). The new annotated chloroplast genome sequence was deposited in GenBank (Accession No. MZ286396).

The chloroplast genome sequence of *Trapa acornis* was 155,538 bp in length, consisting of a small single copy (SSC) region of 18,275 bp, a large single copy (LSC) region of 88,492bp, and two inverted repeat (IR) regions of 24,386 bp. The GC contents of the SSC, LSC, IR are 30.2%, 34.2%, 42.8%, respectively, with the overall content of 36.4%, which is extremely similar to other *Trapa* species. The chloroplast genome encoded a total of 130 genes (85 protein-coding, 37 tRNA, 8 rRNA), with 19 duplicated genes (8 protein-coding, 7 tRNA, 4 rRNA). Intron-exon structure analysis indicated that three protein-coding genes (*clp*P, *ycf*3, and *rps*12) had two introns and seven protein-coding genes (*ndh*B*, ndh*A*, rpl*16*, pet*D, *pet*B*, rpo*C1, and *atp*F) had one intron.

Phylogenomic analysis of *Trapa* and other Lythraceae species was performed using maximum likelihood (ML) method based on the complete chloroplast genomes from six species within *Trapa* and 30 other species within Lythraceae. *Ludwigia octovalvis* (NC031385) was used as an outgroup. ML analysis was conducted using PhyML v.3.0 (Guindon et al. 2010). The phylogenetic tree demonstrated that *T. acornis Nakano* was identified as the sister of *T. bicornis.* It also indicated that *Trapa* was sister to genus *Sonneratia*, which is consistent with previous studies (Huang and Shi 2002; Xue et al. 2017).

## Data Availability

The data that support the findings of this study are openly available in NCBI Genbank with accession code MZ286396 (https://www.ncbi.nlm.nih.gov/nuccore/MZ286396). The associated BioProject, SRA, and Bio-Sample numbers are PRJNA747760, SRR15183333, and SAMN20295638 respectively. Phylogenetic tree using maximum-likelihood (ML) based on plastomes of 35 Lythraceae species with *Ludwigia octovalvis* as an outgroup. Numbers near the nodes represent ML bootstrap values.

## References

[CIT0001] Adkar P, Dongare A, Ambavade S, Bhaskar VH. 2014. *Trapa bispinosa* Roxb: a review on nutritional and pharmacological aspects. Adv Pharmacol Sci. 2014:959813–959830. 10.1155/2014/959830 PMC3941599 24669216

[CIT0002] Bingyang D, Meizhong S, Yang HR, et al. 1996. Nutritional constituents of *Trapa acornis* fruit. J Plant Resour Environ. 5:57–59.

[CIT0003] Chen JR, Ding BY, Funston AM, et al. 2007. Flora of China. Vol. 13. Beijing: Science Press, St. Louis: Missouri Botanical Garden Press, p. 290–291.

[CIT0004] Chorak GM, Dodd LL, Rybicki N, Ingram K, Buyukyoruk M, Kadono Y, Chen YY, Thum RA. 2019. Cryptic Introduction of Water Chestnut (*Trapa*) in the Northeastern United States. Aquat Bot. 155:32–37.

[CIT0005] Dierckxsens N, Mardulyn P, Smits G, et al. 2017. NOVOPlasty: de novo assembly of organelle genomes from whole genome data. Nucleic Acids Res. 45:18. 10.1093/nar/gkw955 PMC5389512 28204566

[CIT0006] Guindon S, Dufayard J-F, Lefort V, Anisimova M, Hordijk W, Gascuel O. 2010. New algorithms and methods to estimate maximum-likelihood phylogenies: assessing the performance of PhyML 3.0. Syst Biol. 59(3):307–321. 20525638 10.1093/sysbio/syq010

[CIT0007] Guo Y, Wu R, Sun G, et al. 2017. Neolithic cultivation of water chestnuts (*Trapa* L.) at Tianluoshan (7000–6300 cal BP), Zhejiang Province, China. Sci Rep. 7:1–8. 29176707 10.1038/s41598-017-15881-w PMC5701232

[CIT0008] Huang Y, Shi S. 2002. Phylogenetics of Lythraceae Sensu Lato: a preliminary analysis based on chloroplast *rbc*L gene, *psa*A-*ycf*3 spacer, and nuclear rDNA internal transcribed spacer (ITS) sequences. Int J Plant Sci. 163(2):215–225.

[CIT0009] Hummel M, Kiviat E. 2004. Review of world literature on water chestnut with implications for management in North America. Journal of Aquatic Plant Management. 42:17–27.

[CIT0412] Jin, J., Yu, W., Yang, J., Song, Y., dePamphilis, C., Yi, T., & Li, D. 2018. GetOrganelle: a fast and versatile toolkit for accurate de novo assembly of organelle genomes. doi: 10.1101/256479 PMC7488116 32912315

[CIT0012] Lim TK. 2013. *Trapa natans*. In: Edible medicinal and non-medicinal plants. Dordrecht: Springer, p. 195–201.

[CIT0013] Takano A, Kadono Y. 2005. Allozyme variations and classification of *Trapa* (Trapaceae) in Japan. Aquat Bot. 83(2):108–118.

[CIT0014] Xue Z-Q, Xue J-H, Victorovna KM, Ma K-P. 2017. The complete chloroplast DNA sequence of *Trapa maximowiczii* Korsh. (Trapaceae), and comparative analysis with other myrtales species. Aquat Bot. 143:54–62.

